# Engineering Small HOMO–LUMO Gaps in Polycyclic Aromatic Hydrocarbons with Topologically Protected States

**DOI:** 10.1021/acs.nanolett.4c01476

**Published:** 2024-04-17

**Authors:** Kaitlin Slicker, Aidan Delgado, Jingwei Jiang, Weichen Tang, Adam Cronin, Raymond E. Blackwell, Steven G. Louie, Felix R. Fischer

**Affiliations:** # Department of Chemistry, University of California, Berkeley, Berkeley, California 94720, United States; ‡ Department of Physics, University of California, Berkeley, Berkeley, California 94720, United States; § Materials Sciences Division, Lawrence Berkeley National Laboratory, Berkeley, California 94720, United States; ∥ Kavli Energy NanoSciences Institute at the University of California, Berkeley, and the Lawrence Berkeley National Laboratory, Berkeley, California 94720, United States; ⊥ Bakar Institute of Digital Materials for the Planet, Division of Computing, Data Science, and Society, University of California, Berkeley, Berkeley, California 94720, United States

**Keywords:** polycyclic aromatic hydrocarbons, nanographene, symmetry protected topological states, small HOMO−LUMO gaps, topological engineering, zero modes

## Abstract

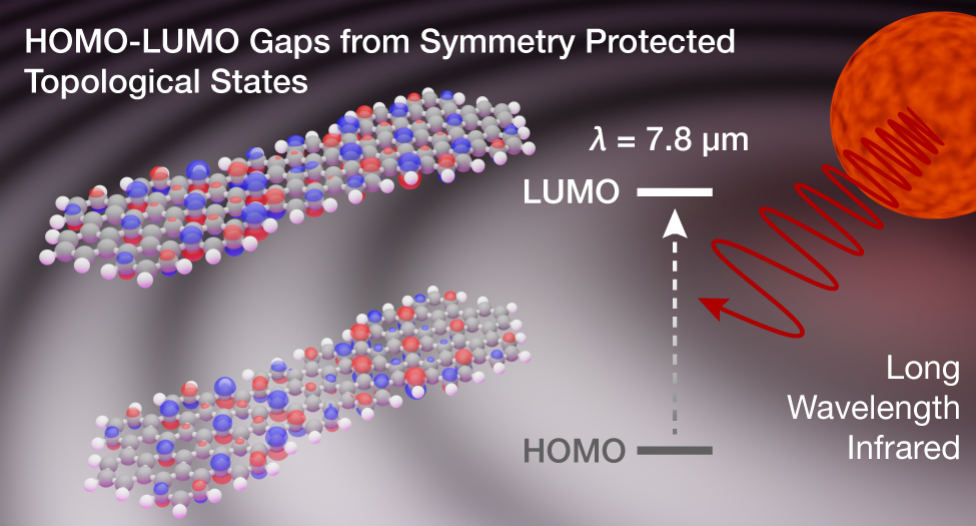

Topological phases in laterally confined low-dimensional nanographenes have emerged as versatile design tools that can imbue otherwise unremarkable materials with exotic band structures ranging from topological semiconductors and quantum dots to intrinsically metallic bands. The periodic boundary conditions that define the topology of a given lattice have thus far prevented the translation of this technology to the quasi-zero-dimensional (0D) domain of small molecular structures. Here, we describe the synthesis of a polycyclic aromatic hydrocarbon (PAH) featuring two localized zero modes (ZMs) formed by the topological junction interface between a trivial and nontrivial phase within a single molecule. First-principles density functional theory calculations predict a strong hybridization between adjacent ZMs that gives rise to an exceptionally small HOMO–LUMO gap. Scanning tunneling microscopy and spectroscopy corroborate the molecular structure of 9/7/9-double quantum dots and reveal an experimental quasiparticle gap of 0.16 eV, corresponding to a carbon-based small molecule long-wavelength infrared (LWIR) absorber.

Topological insulators (TIs) are representatives of an exotic class of quantum materials whose bulk interior retains characteristics of a classical insulator while their surfaces host robust highly conductive in-gap states.^1−3^ While most research has focused on 2D^4−6^ and 3D^7−10^ TIs, recent theoretical and experimental advancements have demonstrated the emergence of symmetry-protected topological phases in graphene nanoribbons (GNRs)—a family of quasi-1D carbon-based nanomaterials.^11,12^ The advent of surface-assisted bottom-up synthesis techniques^13^ has established topological design as a highly versatile route for engineering discrete lattices of zero-mode states that have given rise to tunable bandgaps,^13−15^ locally confined quantum dots,^16^ and robust metallic band structures.^17−19^ The functional translation of this technology to the field of small molecule polycyclic aromatic hydrocarbons (PAHs) has remained elusive. Rather than emulating traditional tools that have been broadly used to shape the electronic structure of closed shell nanographenes, e.g., size, shape, length of conjugation or position, and density of substitutional heteroatom dopants,^20−22^ topological engineering represents a truly complementary and thus far untapped resource in the molecular synthesis toolbox.

The topological character of a laterally confined nanographene is determined by the symmetry of the terminating crystallographic unit cell expressed by a  invariant (trivial topology, , nontrivial topology, )^11,23^ or the generalized  invariant ( is an integer).^12^ The bulk-boundary correspondence, a guiding principle in topological matter, dictates that the interface between two topologically distinct unit cells gives rise to localized symmetry-protected topological ZMs.^11,24^ We have previously shown that the controlled hybridization of adjacent ZMs can give rise to topological semiconductors, metallic bands, and quantum dots embedded within the periodic lattice of a GNR.^25,26^Figure 1a shows a schematic representation of a superlattice of short segments of 7-armchair GNRs (7-AGNRs) and 9-armchair GNRs (9-AGNRs) grown from a single molecular precursor (DBBT in Figure 1a), featuring a pattern of topological trivial () and nontrivial () unit cells, respectively. The topological interface between 7- and 9-AGNR segments gives rise to a localized half-filled interface state hosted alternately on the A or B sublattice of graphene (conceptually represented by red and blue circles). The hybridization between these 7/9 interface states depends among others on the distance and the orbital overlap of the contributing localized interface wave functions and can be described within a tight-binding model using the intra- and intercell hopping amplitudes *t*_intra_ and *t*_inter_, respectively.^27^ While a similar analysis has been applied to the exploration of the interaction of nearby isolated topological interface states within single otherwise uniform ribbons, e.g., topological 7/9 quantum dots hosted within an extended GNR, the application of this model to molecular 0D structures requires further adaptation.

**Figure 1:**
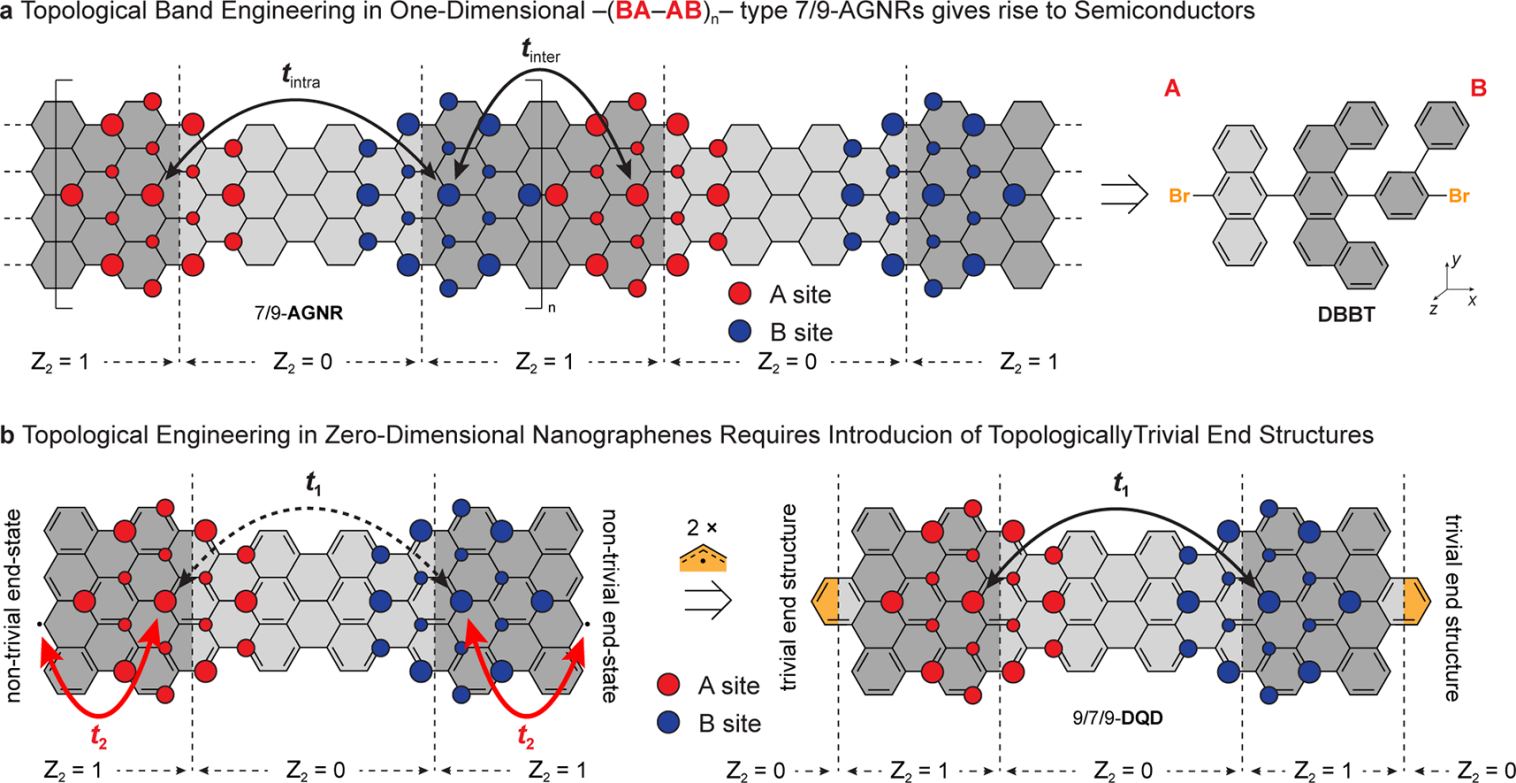
Topological engineering in 1D and 0D nanographenes. (a) The introduction of a superlattice of topological interface states in semiconducting 7/9-AGNRs gives rise to a pair of symmetry protected topological bands that depend on the intra- and intercell hopping parameters *t*_intra_ and *t*_inter_, respectively. (b) Nontrivial zigzag end-states in 0D nanographenes hybridize (via *t*_2_) with 7/9-junction states to add to the interaction (*t*_1_) between topological interface states, leading to a 4-state complex. Termination of the zigzag edges with allyl radicals eliminates the topological zigzag end-states and recovers a pure hybridization (*t*_1_) between the two 7/9-junction states. Red and blue circles are a conceptual representation of the localization of the 7/9-junction state on the A and B sublattices.

Here we report the design and on-surface synthesis of a discrete polycyclic aromatic hydrocarbon (PAH) featuring frontier orbitals that are formed by the hybridization of two adjacent topologically protected junction states. Figure 1b shows the conceptual translation of the topological 7/9-heterojunction into the structure of a small molecule. A short segment corresponding to three topological unit cells with  () of a 7-AGNR is flanked on either side by only two unit cells with  () derived from a 9-AGNR. This arrangement in principle would give rise to two adjacent topological 7/9-junction states reminiscent of the unit cell in 7/9-AGNRs. In this case, however, both ends of the PAH are lined by a nontrivial termination, a 9-AGNR unit cell with  (), giving rise to a second pair of topological end-states as the tetracene unit cell of 9-AGNR borders vacuum  (). The hybridization between the 7/9-junction states and the adjacent end-state is stronger than the coupling across the short 7-AGNR segment (*t*_2_ ≫ *t*_1_), leading to the opening of a sizable HOMO–LUMO gap.^28^ In order to decouple the interaction of the 7/9-interface state with end-states, we quenched the nontrivial 9-AGNR unit cell on either end of the PAH with an allyl radical. The resulting termination, structurally similar to a hexabenzocoronene fragment, is trivial with  (thereby eliminating the topological end state) and effectively retaining only the weaker interaction (*t*_1_) between the topological 7/9-interface states. Atomically precise molecular 9/7/9-double quantum dots (9/7/9-DQDs) were synthesized from molecular precursors on a Au(111) surface and characterized in ultrahigh vacuum (UHV) by using cryogenic scanning tunneling microscopy (STM) and spectroscopy (STS). Experimental results are corroborated by first-principles calculations, revealing a HOMO–LUMO gap of ∼0.16 eV. We herein demonstrate that topological engineering can give access to discrete organic chromophores with HOMO–LUMO transitions spanning the long wavelength to the far-infrared region of the electromagnetic spectrum largely dominated by inorganic semiconductors like InSb, PbSe, and Hg_1–*x*_Cd_*x*_Te^29−32^ far beyond the reaches of conventional PAHs.^33−39^

The synthesis of the common molecular precursor **1** that gives rise to both 7/9-QDs and 9/7/9-DQDs is depicted in Scheme 1. Acid-catalyzed condensation of 7-phenylnapthalne-2-ol (**2**) with [1,1′-biphenyl]-4-carbaldehyde (**3**) gave 14-([1,1′-biphenyl]-4-yl-2,12-diphenyl-14*H*-dibenzo[*a*,*j*]xanthene (**4**) in 96% yield. Oxidation of the benzylic methine group in **4** with PbO_2_ in AcOH gave xanthenol **5**. Protonation of the hydroxyl group followed by elimination of water gave pyrilium salt **6** in 82% yield. Condensation of **6** with the sodium salt of 2-(10-bromoanthracen-9-yl)acetic acid (**7**) gave the benzo[*m*]tetraphene core **1** as the molecular building block for 7/9-QDs and 9/7/9-DQD as a minor component in the product mixture. Analytically pure samples of **1** suitable for surface deposition were obtained by fractional recrystallization from CHCl_3_.

**Scheme 1:**
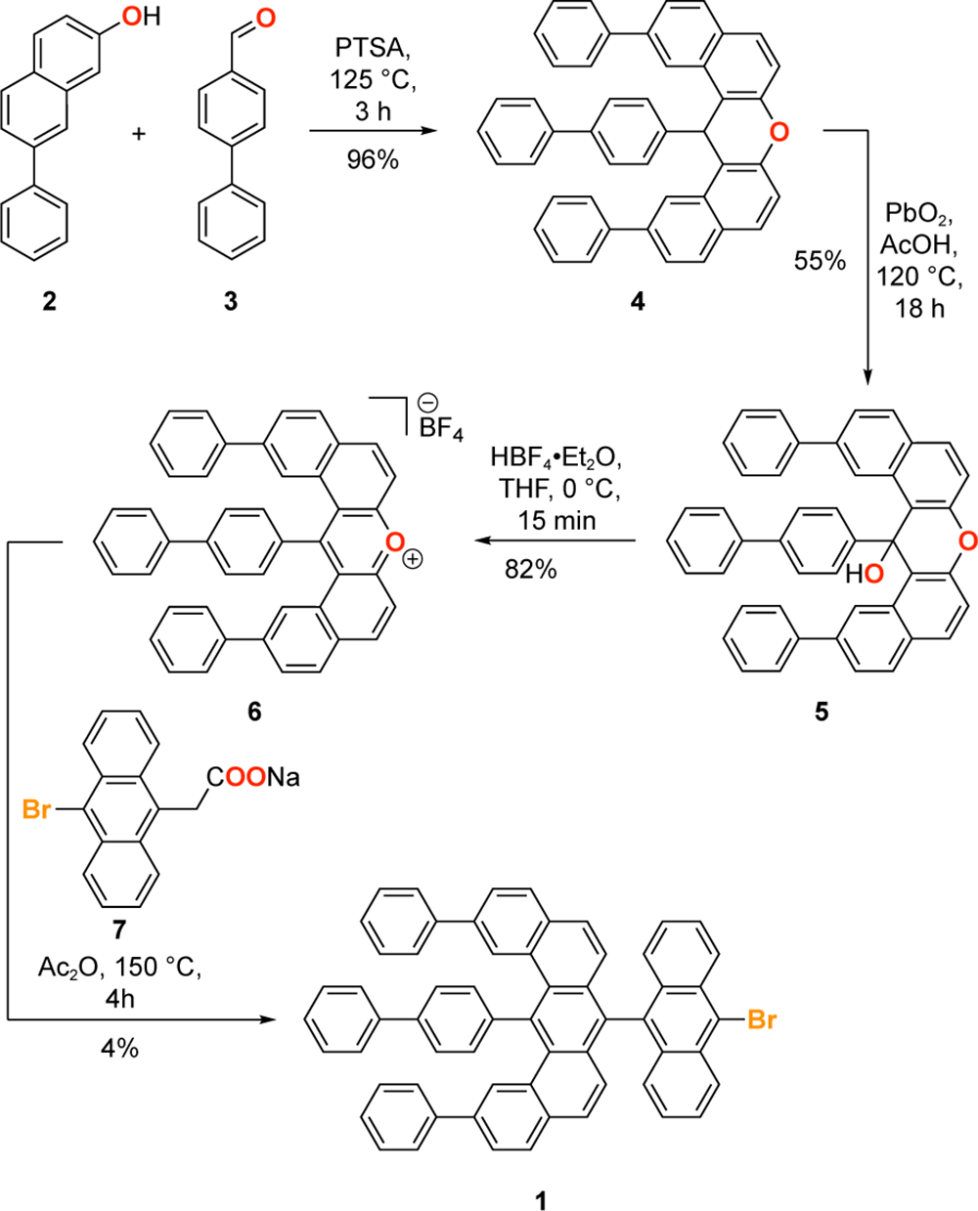
Synthesis of Molecular Precursor **1** for 7/9-QD and 9/7/9-DQD

Samples of 7/9-QD and 9/7/9-DQD for cryogenic (4 K) scanning tunneling microscopy (STM) imaging were prepared following established surface deposition techniques.^13^ Initial attempts to sublime **1** in UHV from a Knudsen cell evaporator (*T* = 460 K) onto a Au(111) surface revealed only decomposition products characterized by the cleavage of the labile 9-bromoanthracenyl group (Supporting Information Figure S1a–c). To circumvent this thermal decomposition, samples of **1** dispersed in an inert matrix of pyrene were deposited on Au(111) surfaces using matrix-assisted direct (MAD) transfer protocols (Figure 2a).^40,41^ Molecule-decorated surfaces were annealed to 353 K for 10 h to facilitate lateral diffusion and traceless sublimation of the bulk pyrene matrix. Further annealing at 673 K for 30 min induces the surface-assisted homolytic cleavage of C–Br bonds, dimerization of intermediate surface stabilized radicals, and thermal cyclodehydrogenation that gives rise to the extended π-system in the fused QDs. Large area topographic STM images recorded on annealed MAD samples of **1** predominately show isolated 7/9-QDs sparsely interspersed with 9/7/9-DQDs (Figure S2a,b). Figure 2b shows a low-bias topographic STM image of a 9/7/9-DQD featuring atomically smooth edges with an apparent height, width, and length of 0.195 ± 0.02, 1.39 ± 0.04, and 3.54 ± 0.02 nm, respectively.

**Figure 2:**
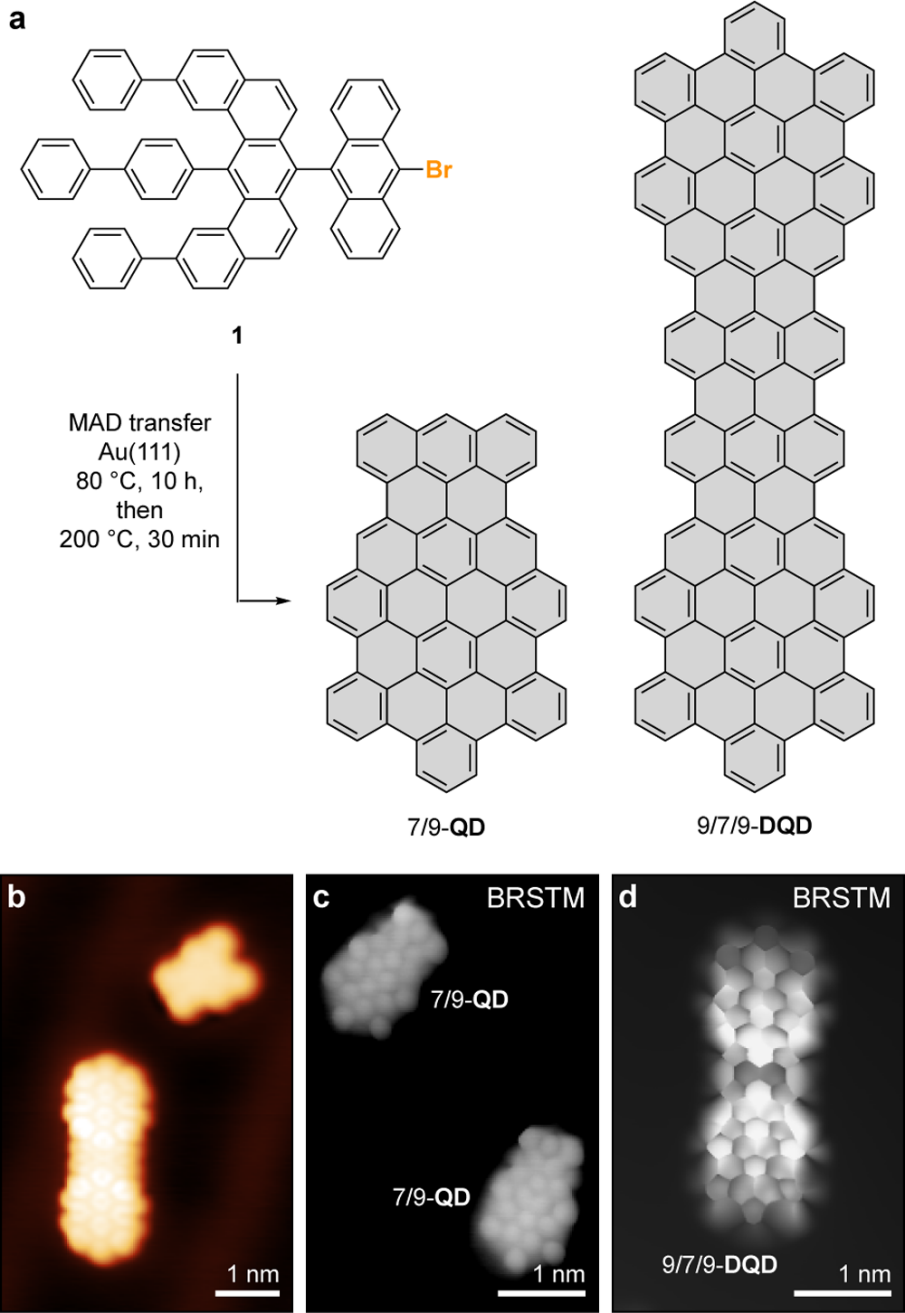
Surface-assisted assembly of 7/9-QD and 9/7/9-DQD. (a) Schematic representation of the MAD transfer followed by thermally induced dimerization and cyclodehydrogenation of **1** that gives rise to 7/9-QD and 9/7/9-DQD. (b) STM topographic image of a 9/7/9-DQD along with a fragment of a 7/9-QD missing a phenyl ring following the thermal cyclodehydrogenation at 673 K (*V*_s_ = 0.05 V, *I*_t_ = 100 pA). (c) BRSTM image of two isolated 7/9-QD showing the internal bonding of the 7/9 topological junction interface (*V*_s_ = 0.01 V, *I*_t_ = 300 pA). (d) BRSTM image of a representative 9/7/9-DQD showing the signature of a low-lying topological state (*V*_s_ = −0.02 V, *I*_t_ = 100 pA).

Tip-functionalized (CO) bond-resolved STM (BRSTM) images recorded on a representative sample of 7/9-QD (Figure 2c) show the characteristic internal bonding associated with the topological interface defined by the laterally fused anthracene (7-AGNR segment) and tetracene (9-AGNR segment) units. BRSTM images recorded on 9/7/9-DQD (Figure 2d; see Figure S2c for Laplace filtered images) samples show the internal bonding associated with the dimerization and cyclodehydrogenation of **1** giving rise to two adjacent topological interface states. The apparent bond distortion in BRSTM images of 9/7/9-DQDs is the signature of a low-bias state emerging from the interaction of two adjacent 7/9 topological interface states, absent in samples of the 7/9-QD.

Having resolved the chemical structure of 7/9-QD and 9/7/9-DQD, we shifted our focus to the characterization of their electronic structure using differential tunneling spectroscopy. Figure 3a shows typical d*I*/d*V* point spectra for 7/9-QDs recorded with a CO-functionalized STM tip at the positions highlighted in the inset. Irrespective of the placement of the tip, spectra of 7/9-QDs are featureless in the range between −1.0 V < *V*_s_ < +1.0 V against the Au(111) background. This behavior indicates some significant interaction of the HOMO and LUMO states with the Au substrate, making the identification of the molecular orbital levels in this energy range difficult (Figure S3). In contrast, d*I*/d*V* point spectra recorded at the highlighted positions above a 9/7/9-DQD molecule show characteristic signatures of low-bias states (Figure 3b). Four distinct features can be observed in the range between −1.50 V < *V*_s_ < +1.50 V. Two shoulders at *V*_s_ = +1.35 V (peak 1) and *V*_s_ = −1.20 V (peak 4) along with a broad peak at *V*_s_ = +0.16 V (peak 2) and a much smaller spectral feature close to *V*_s_ = 0.00 V (peak 3) dominate the spectrum (Figure S4). The relative signal intensities of peaks 1 and 3 are largest when the STM tip is placed at the position of the 7/9-junction interface (orange line in Figure 2B). While peak 2 is prominently featured in d*I*/d*V* spectra recorded along the 7-armchair edge of the fused bisanthene segment (red line in Figure 3b), the intensity of peak 3 is barely detectable in spectra recorded at either the 7- or the 9-armchair edge of the 9/7/9-DQD.

**Figure 3:**
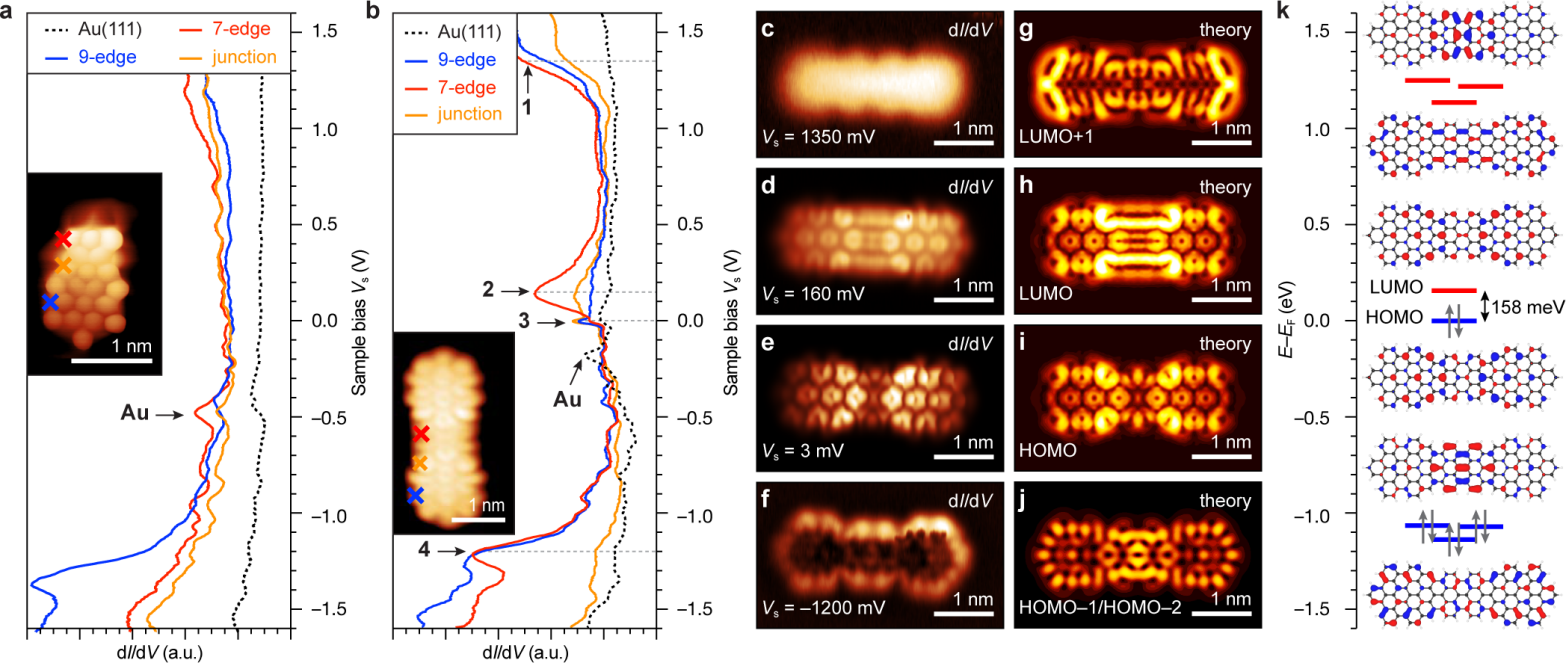
Electronic structure characterization of 7/9-QD and 9/7/9-DQD. (a) STS d*I*/d*V* spectra recorded on a 7/9-QD (spectroscopy: *V*_ac_ = 10 mV, *f* = 455 Hz; imaging: *V*_s_ = 0.01 V, *I*_t_ = 400 pA, CO-functionalized tip) and (b) on a 9/7/9-DQD (spectroscopy: *V*_ac_ = 10 mV, *f* = 455 Hz; imaging: *V*_s_ = 0.05 V, *I*_t_ = 100 pA, CO-functionalized tip). (c–f) Constant-height d*I*/d*V* maps recorded at the indicated biases (spectroscopy: *V*_ac_ = 10 mV, *f* = 455 Hz). (g–j) Projections of the LDOS calculated within the DFT approximation evaluated at the energies corresponding to the LUMO+1, LUMO, HOMO, and HOMO–1/HOMO–2. (k) DFT calculated molecular orbital diagram for 9/7/9-DQD. The energy of the HOMO has been calibrated to the *E*_F_.

Differential conductance maps recorded over a bias range of +0.5 V < *V*_s_ < +1.5 V are largely featureless and only show a diffuse intensity along the center of a 9/7/9-DQD (Figure 3c and Figure S4). The signal intensity in d*I*/d*V* maps across a bias of −1.3 V < *V*_s_ < −0.6 V shows a halo of nodal patterns lining the edges of 9/7/9-DQD juxtaposed by a dark featureless center. In stark contrast, differential conductance maps recorded at *V*_s_ = 160 mV (peak 2, Figure 3d) and *V*_s_ = +3 mV (peak 3, Figure 3e) show a unique pattern of nodes. The highest signal intensity coincides with the position of the two 7/9-junctions. The wave function patterns associated with peak 2 (Figure 3d) and peak 3 (Figure 3e) are reminiscent of an antibonding and bonding linear combination of molecular orbitals emerging from the interaction of two topologically protected 7/9-interface states, respectively.

We further explored the electronic structure of 7/9-QD and 9/7/9-DQD using ab initio DFT.^42,43^ A molecular orbital diagram for the singlet ground state of the smaller 7/9-QD is depicted in Figure S3. The interaction between the topological 7/9-interface state and the nontrivial 7-AGNR end-state in 7/9-QDs (both half-filled) leads to the opening of a sizable HOMO–LUMO gap (*E*_g,DFT_ = 0.872 eV) in the theoretical results indicative of a strongly hybridized system. Figure 3k shows the molecular orbital diagram and orbital wave functions for the singlet ground state of 9/7/9-DQD calculated using the DFT approximation. The theoretically predicted quasiparticle HOMO–LUMO gap is small, *E*_g,DFT_ = 0.158 eV, corresponding to a long-wave infrared (LWIR) excitation of λ = 7.8 μm (367 K blackbody radiation maximum). Projections of the local density of states (LDOS) evaluated at the energies of LUMO (*E* – *E*_F_ = 0.16 eV) and HOMO (*E* – *E*_F_ = 0.00 eV) (Figures 3h,i) faithfully reproduce the nodal patterns observed in the corresponding d*I*/d*V* maps (Figures 3d,e). Both frontier molecular orbitals are localized to the 7/9-junction interface and are dominated by contributions from a bonding (LUMO) and an antibonding (HOMO) linear combination of the two adjacent topological states. Similar LDOS projections at higher and lower energies corresponding to the LUMO+1 (*E* – *E*_F_ = 1.14 eV) and a mixture of the energetically very similar HOMO–1/HOMO–2 (*E* – *E*_F_ = −1.07 eV) are shown in Figures 3g,j. d*I*/d*V* maps qualitatively reproduce the LDOS distribution calculated for the LUMO+1 and the HOMO–1. Figures 3c and 3g show the highest signal intensity along the backbone of the 9/7/9-DQD π-system, while Figures 3f and 3j feature two prominent protrusions lining the edges of the 7-AGNR segment contrasted by a reduced intensity along the 9-AGNR backbone. The correspondence between theoretical LDOS projections and experimental d*I*/d*V* maps supports the assignment of the spectral features corresponding to peak 2 and peak 3 in Figure 3b to the LUMO and HOMO states of 9/7/9-DQD (the antibonding and bonding pair of the topological interface states), giving rise to an experimental HOMO–LUMO gap of *E*_g,exp_ ∼ 0.16 eV.

We herein demonstrate the concept of topological engineering as a fully complementary and, thus far, untapped resource in the design and bottom-up synthesis of functional 0D nanographenes. Our strategy builds on the controlled spatial arrangement and hybridization of topological 7/9-interface states that give rise to a highly localized pair of frontier orbitals. STM imaging and differential conductance spectroscopy on Au(111) reveal an exceptionally small HOMO–LUMO bandgap of 0.16 eV, corresponding to LWIR absorption. First-principles DFT calculations corroborate the topological character of the frontier states in 9/7/9-DQD. The access to topologically engineered ultralow-bandgap nanographenes paves the way toward the realization IR-sensitized photovoltaics and LWIR detectors based on carbon nanotechnology.

## Supporting Information Available

Computational and experimental details, STM/BRSTM images, additional spectroscopic data on 9/7/9-DQD, calculations, and ^1^H and ^13^C NMR spectra for all compounds (PDF)
