# Supplementary material for: Engineering Small HOMO–LUMO Gaps in Polycyclic Aromatic Hydrocarbons with Topologically Protected States
Source: Nano Lett. 2024 Apr 17;24(17):5387–92. doi: 10.1021/acs.nanolett.4c01476 (PMC11066967; doi:10.1021/acs.nanolett.4c01476)
Supplement: Supplementary file 1 — nl4c01476_si_001.pdf [file nl4c01476_si_001.pdf]

Engineering Small HOMO-LUMO Gaps in Polycyclic Aromatic Hydrocarbons with Topologically Protected States

Kaitlin Slicker,[†,‡] *Aidan Delgado,*[†,‡] *Jingwei Jiang,*[‡,§,‡] *Weichen Tang,*[‡] *Adam Cronin,*[†] *Raymond E. Blackwell,*[†] *Steven G. Louie,*[‡,§,*] *Felix R. Fischer*[†,§,¶,◊,*]

[†]Department of Chemistry, University of California, Berkeley, CA 94720, U.S.A.

[‡]Department of Physics, University of California, Berkeley, CA 94720, U.S.A.

[§]Materials Sciences Division, Lawrence Berkeley National Laboratory, Berkeley, CA 94720, U.S.A.

[¶]Kavli Energy NanoSciences Institute at the University of California Berkeley and the Lawrence Berkeley National Laboratory, Berkeley, California 94720, U.S.A.

[◊]Bakar Institute of Digital Materials for the Planet, Division of Computing, Data Science, and Society, University of California, Berkeley, CA 94720, USA.

1.	**Figure S1.** (a) STM topographic image of a sample of 1 as sublimed onto a Au(111) surface (V_s = 0.05 V, I_t = 100 pA). (b) Representative BRSTM image of a fragment of 1 showing prevalent loss of anthracene fragments under the sublimation conditions (V_s = 0.01 V, I_t = 230 pA). (c) Schematic representation of the chemical structure of the fragment in (b).	S2
2.	**Figure S2.** Large area topographic STM images recorded on an annealed MAD sample of 1. (a,b) STM images predominately show isolated 7/9-QDs sparsely interspersed with 9/7/9-DQDs highlighted by white ovals (V_s = 0.05 V, I_t = 20 pA). (c) Laplace filtered BRSTM image of a representative 9/7/9-DQD showing the signature of a low-lying topological state (V_s = –0.02 V, I_t = 100 pA).	S3
3.	**Figure S3.** DFT calculated molecular orbital diagram for 7/9-QD. The energy of the HOMO has been calibrated to the E_F.	S4
4.	**Figure S4.** Electronic structure characterization of 9/7/9-DQD. (a) STS d*I*/d*V* spectra recorded on a 9/7/9-DQD (spectroscopy: V_{ac} = 10 mV, *f* = 455 Hz; imaging: V_s = 0.05 V, I_t = 100 pA, CO-functionalized tip). (b–i) Constant height d*I*/d*V* maps recorded at the indicated biases (spectroscopy: V_{ac} = 10 mV, *f* = 455 Hz).	S5
5.	**Computational Details**	S6
6.	**Materials and Instrumentation**	S6
7.	**Figure S5.** ^{1}H NMR (600 MHz, CD_2Cl_2) of 14-([1,1'-biphenyl]-4-yl)-2,12-diphenyl-14*H*-dibenzo[*a,j*]xanthene (**4**) at 24 °C.	S7
8.	**Figure S6.** ^{13}C{^{1}H} NMR (151 MHz, CD_2Cl_2) spectrum of 14-([1,1'-biphenyl]-4-yl)-2,12-diphenyl-14H-dibenzo[a,j]xanthene (**4**) at 24 °C.	S8
9.	**Figure S7.** ^{1}H NMR (600 MHz, CD_2Cl_2) of 14-([1,1'-biphenyl]-4-yl)-2,12-diphenyldibenzo[*a,j*]xanthen-7-ium tetrafluoroborate (**6**) at 24 °C.	S9
10.	**Figure S8.** ^{13}C{^{1}H} NMR (151 MHz, CD_2Cl_2) spectrum of 14-([1,1'-biphenyl]-4-yl)-2,12-diphenyldibenzo[*a,j*]xanthen-7-ium tetrafluoroborate (**6**) at 24 °C.	S10
11.	**Figure S9.** ^{1}H NMR (600 MHz, CD_2Cl_2) of 14-([1,1'-biphenyl]-4-yl)-7-(10-bromoanthracen-9-yl)-2,12 diphenylbenzo[m]tetraphenetetraphene (**1**) at 24 °C.	S11
12.	**Figure S10.** ^{13}C{^{1}H} NMR (151 MHz, CD_2Cl_2) spectrum of 14-([1,1'-biphenyl]-4-yl)-7-(10-bromoanthracen-9-yl)-2,12 diphenylbenzo[*m*]tetraphenetetraphene (**1**) at 24 °C.	S12
13.	**References**	S13

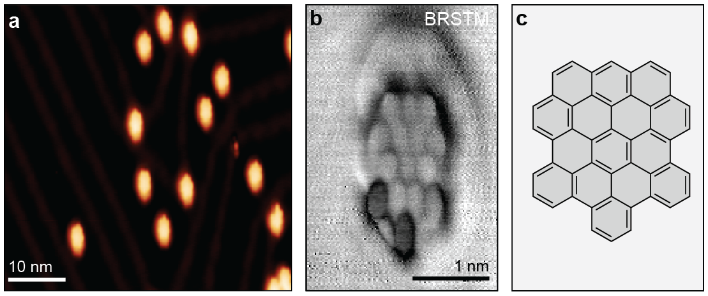

Figure S1. (a) STM topographic image of a sample of 1 as sublimed onto a Au(111) surface (V_s = 0.05 V, I_t = 100 pA). (b) Representative BRSTM image of a fragment of 1 showing prevalent loss of anthracene fragments under the sublimation conditions (V_s = 0.01 V, I_t = 230 pA). (c) Schematic representation of the chemical structure of the fragment in (b).

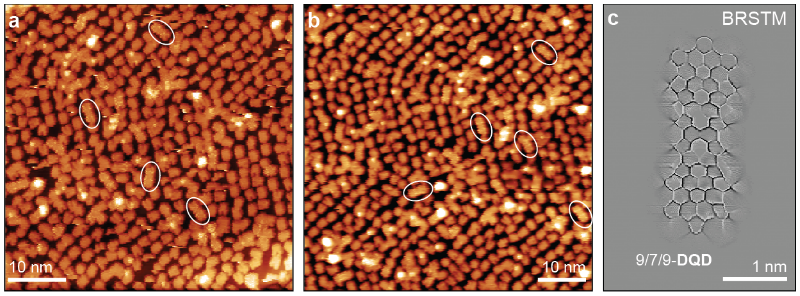

Figure S2. Large area topographic STM images recorded on an annealed MAD sample of 1. (a,b) STM images predominately show isolated 7/9-QDs sparsely interspersed with 9/7/9-DQDs highlighted by white ovals (V_s = 0.05 V, I_t = 20 pA). (c) Laplace filtered BRSTM image of a representative 9/7/9-DQD showing the signature of a low-lying topological state (V_s = –0.02 V, I_t = 100 pA).

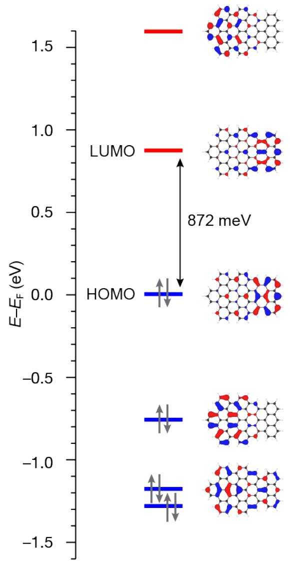

Figure S3. DFT calculated molecular orbital diagram for 7/9-QD in the local density approximation (LDA). The energy of the HOMO has been calibrated to the E_F.

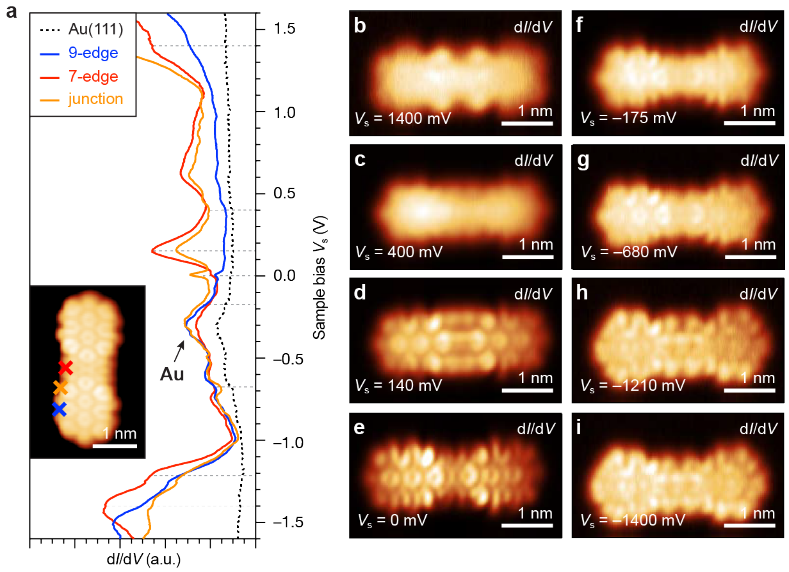

Figure S4. Electronic structure characterization of 9/7/9-DQD. (a) STS dI/dV spectra recorded on a 9/7/9-DQD (spectroscopy: V_{ac} = 10 mV, f = 455 Hz; imaging: V_s = 0.05 V, I_t = 100 pA, CO-functionalized tip). (b–i) Constant height dI/dV maps recorded at the indicated biases (spectroscopy: V_{ac} = 10 mV, f = 455 Hz).

Computational Details. First-principles DFT calculations in the local-density approximation were performed using the Quantum Espresso packages.[1-2] A supercell geometry was employed, with a 10 Å vacuum spacing applied in all directions to prevent interaction between replicas. The atomic geometry was fully relaxed until all components of the forces on each atom were smaller than 0.02 eV/Å. A 100 Ry wavefunction energy cut-off, along with scalar relativistic and norm-conserving pseudopotentials for C and H, were applied.[3-4]

Materials and Instrumentation. Unless otherwise stated, all manipulations of air and/or moisture sensitive compounds were carried out in oven-dried glassware, under an atmosphere of N_2. All solvents and reagents were purchased from Alfa Aesar, Spectrum Chemicals, Acros Organics, TCI America, and Sigma-Aldrich and were used as received unless otherwise noted. Organic solvents were dried by passing through a column of alumina and were degassed by vigorous bubbling of N_2 through the solvent for 20 min. Flash column chromatography was performed on SiliCycle silica gel (particle size 40–63 µm). Thin layer chromatography was carried out using SiliCycle silica gel 60 Å F-254 precoated plates (1.0 mm thick) and visualized by UV absorption. All ^{1}H and ^{13}C{^{1}H} NMR spectra were recorded on Bruker AV-600 and AV-400 MHz spectrometers, and are referenced to residual solvent peaks (CD_2Cl_2 ^{1}H NMR = 5.32 ppm, ^{13}C{^{1}H} NMR = 53.84 ppm). ESI mass spectrometry was performed on a Finnigan LTQFT (Thermo) spectrometer in positive ionization mode.

14-([1,1'-biphenyl]-4-yl)-2,12-diphenyl-14*H*-dibenzo[*a,j*]xanthene (**4**) A 100 mL flask was charged with 7-phenylnaphthalen-2-ol (4.25 g, 19.29 mmol), [1,1'-biphenyl]-4-carbaldehyde (1.74 g, 9.55 mmol), and *p*-TSA monohydrate (0.07 g, 0.37 mmol). The flask was heated to 120 °C for 3 h. The solid was cooled to 24 °C and stirred in a mixture of EtOH/H_2O (1/3) for 1 h. The solid was filtered and washed with EtOH to yield **4** (5.40 g, 9.20 mmol, 96 %) as a colorless solid. ^{1}H NMR (600 MHz, CD_2Cl_2) δ 8.65–8.62 (m, 2H), 7.97 (d, *J* = 8.3 Hz, 2H), 7.91 (d, *J* = 8.9 Hz, 2H), 7.80–7.75 (m, 4H), 7.78–7.73 (m, 2H), 7.71 (dd, *J* = 8.3, 1.7 Hz, 2H), 7.60 (dd, *J* = 8.5, 6.9 Hz, 4H), 7.57 (d, *J* = 8.9 Hz, 2H), 7.55–7.50 (m, 2H), 7.52–7.45 (m, 4H), 7.36 (dd, *J* = 8.5, 7.0 Hz, 2H), 7.32–7.26 (m, 1H), 6.68 (s, 1H) ppm; ^{13}C{^{1}H} NMR (151 MHz, CD_2Cl_2) δ 148.8, 144.5, 141.5, 140.4, 139.8, 139.6, 131.6, 130.2, 129.4, 129.0, 128.8, 128.8, 128.6, 127.7, 127.5, 127.3, 127.2, 126.9, 124.2, 121.0, 118.1, 117.0, 38.1 ppm; HRMS (ESI-TOF) *m*/*z*: $[C_{45}H_{31}O]^+$ calcd. $[C_{45}H_{31}O]$ 587.2375; found 587.2369.

14-([1,1'-biphenyl]-4-yl)-2,12-diphenyldibenzo[*a,j*]xanthen-7-ium tetrafluoroborate (**6**) A 100 mL three neck flask was charged with **4** (0.5 g, 0.85 mmol) and PbO_2 (0.5 g, 2.09 mmol) in AcOH (8.5 mL). The reaction mixture was stirred at 120 °C for 18 h. The reaction mixture was cooled to 24 °C. The solid was filtered, washed with H_2O, and dried under vacuum for 18 h. The crude 14-([1,1'-biphenyl]-4-yl)-2,12-diphenyl-14H-dibenzo[a,j]xanthen-14-ol (**5**) (0.285 g, 0.47 mmol, 55 %) was used without further purification. An oven dried 50 mL three neck flask was charged with **5** (0.136 g, 0.23 mmol) in anhydrous THF (3 mL) under N_2. The brown suspension was cooled to 0 °C and HBF_4 (0.2 mL, 2.3 mmol, 50-55 wt.% in Et_2O) was added dropwise and stirred for 15 min. At 0 °C Et_2O was added, the red precipitate was filtered and washed with Et_2O yielding **6** (0.125 g, 0.19 mmol, 82 %) as a red solid. ^{1}H NMR (600 MHz, CD_2Cl_2) δ 8.90 (d, *J* = 9.0 Hz, 2H), 8.34 (d, *J* = 8.6 Hz, 4H), 8.27 (d, *J* = 7.9 Hz, 2H), 8.10 (dd, *J* = 8.1, 1.6 Hz, 2H), 7.88–7.85 (m, 2H), 7.82 (dd, *J* = 8.4, 6.5 Hz, 4H), 7.67 (dt, *J* = 25.9, 7.3 Hz, 3H), 7.32–7.25 (m, 6H), 7.21 (t, *J* = 7.5 Hz, 4H).ppm; ^{13}C{^{1}H} NMR (151 MHz, CD_2Cl_2) δ 166.4, 159.8, 146.9, 145.28, 143.3, 139.1, 138.6, 137.6, 132.3, 131.8, 130.9, 129.6, 129.3, 129.1, 129.1, 128.7, 128.6, 127.9, 127.5, 127.4, 127.1, 122.2, 117.0 ppm; HRMS (ESI-TOF) *m*/*z*: $[C_{45}H_{31}O]^+$ calcd. $[C_{45}H_{31}O]$ 585.2218; found 585.2213.

14-([1,1'-biphenyl]-4-yl)-7-(10-bromoanthracen-9-yl)-2,12 diphenylbenzo[*m*]tetraphenetetraphene (**1**) An oven dried 50 mL Schlenk flask was charged with **6** (168 mg, 0.25 mmol) and sodium 2-(10-bromoanthracen-9-yl)acetate (253 mg, 0.75 mmol) in freshly distilled, degassed Ac_2O under N_2. The suspension was placed in a 150 °C preheated oil bath and stirred for 3.5 h. The solution was cooled to 24 °C, MeOH was added, and the solution was filtered and washed with MeOH. Column chromatography (SiO_2; 1:3 DCM/hexanes) yielded a light yellow solid. The crude solid was recrystallized from $CHCl_3$ to yield **1** (9.4 mg, 0.011 mmol, 4 %) as colorless crystals. ^{1}H NMR (600 MHz, CD_2Cl_2) δ 8.79 (d, *J* = 9.0 Hz, 2H), 8.09 (d, 8.1 Hz, 2H), 7.98 (s, 2H), 7.92 (d, 8.1 Hz, 2H), 7.83 (t, *J* = 7.6 Hz, 4H), 7.70 (dd, *J* = 8.0, 1.7 Hz, 2H), 7.71–7.62 (m, 4H), 7.59–7.53 (m, 1H), 7.45–7.38 (m, 4H), 7.30 (ddd, *J* = 8.8, 6.3, 1.1 Hz, 2H), 7.27–7.22 (m, 4H), 7.24–7.14 (m, 6H), 6.92 (d, *J* = 9.0 Hz, 2H), 1.55 (s, 17H), 0.12 (s, 8H) ppm; ^{13}C{^{1}H} NMR (151 MHz, CD_2Cl_2) δ 145.3, 142.1, 141.4, 140.7, 138.9, 137.4, 135.1, 133.6, 133.1, 133.0, 132.9, 132.3, 132.0, 131.0, 130.2, 129.4, 129.1, 129.0, 129.0, 128.9, 128.5, 128.4, 128.3, 127.8, 127.8, 127.8, 127.7, 127.4, 126.9, 125.9, 125.2, 124.1 ppm; HRMS (ESI-TOF) *m*/*z*: $[C_{60}H_{37}Br]^+$ calcd. $[C_{60}H_{37}Br]$ 836.2079; found 836.2068.

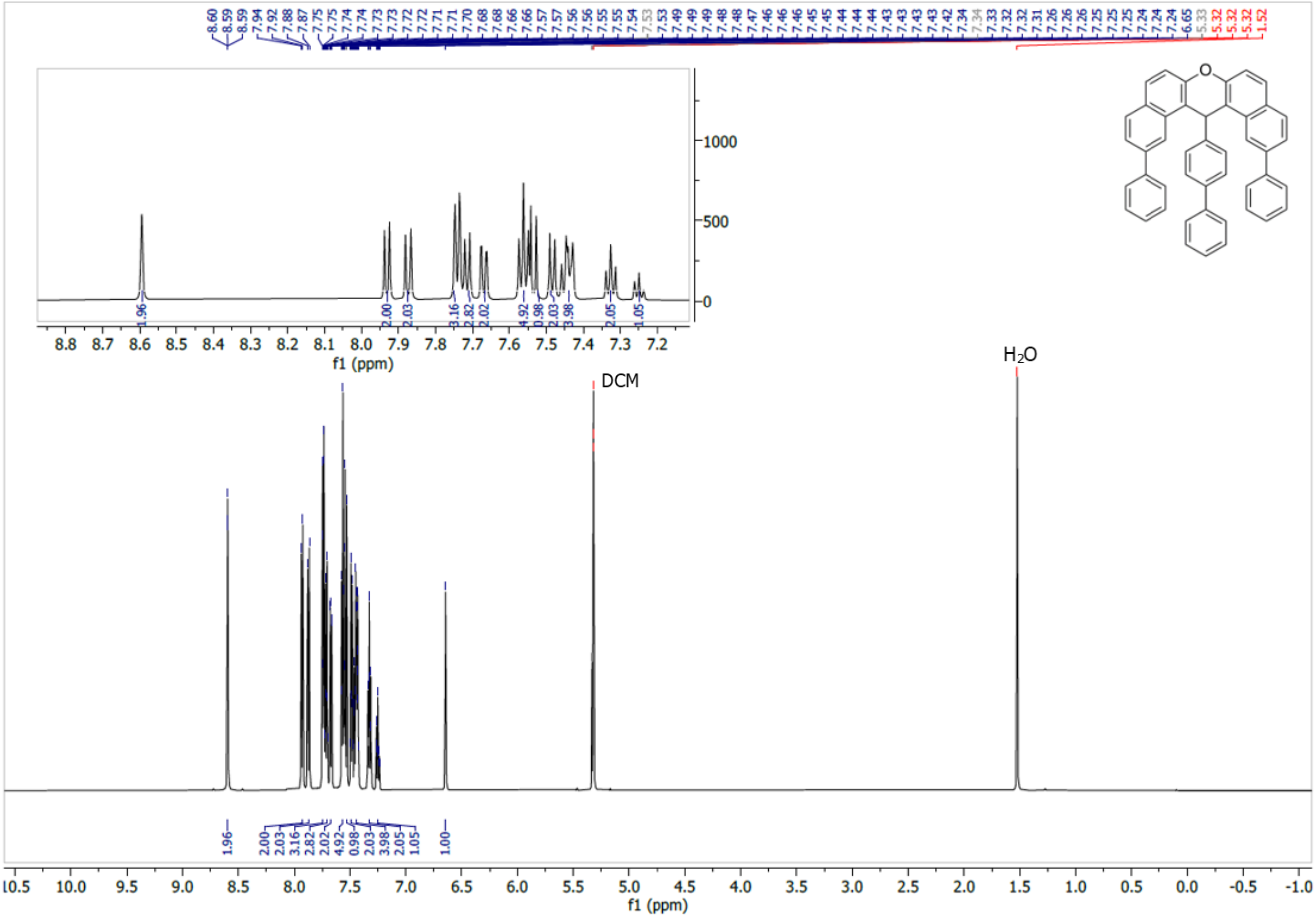

Figure S5. ^{1}H NMR (600 MHz, CD_2Cl_2) of 14-([1,1'-biphenyl]-4-yl)-2,12-diphenyl-14*H*-dibenzo[*a*,*j*]xanthene (**4**) at 24 °C.

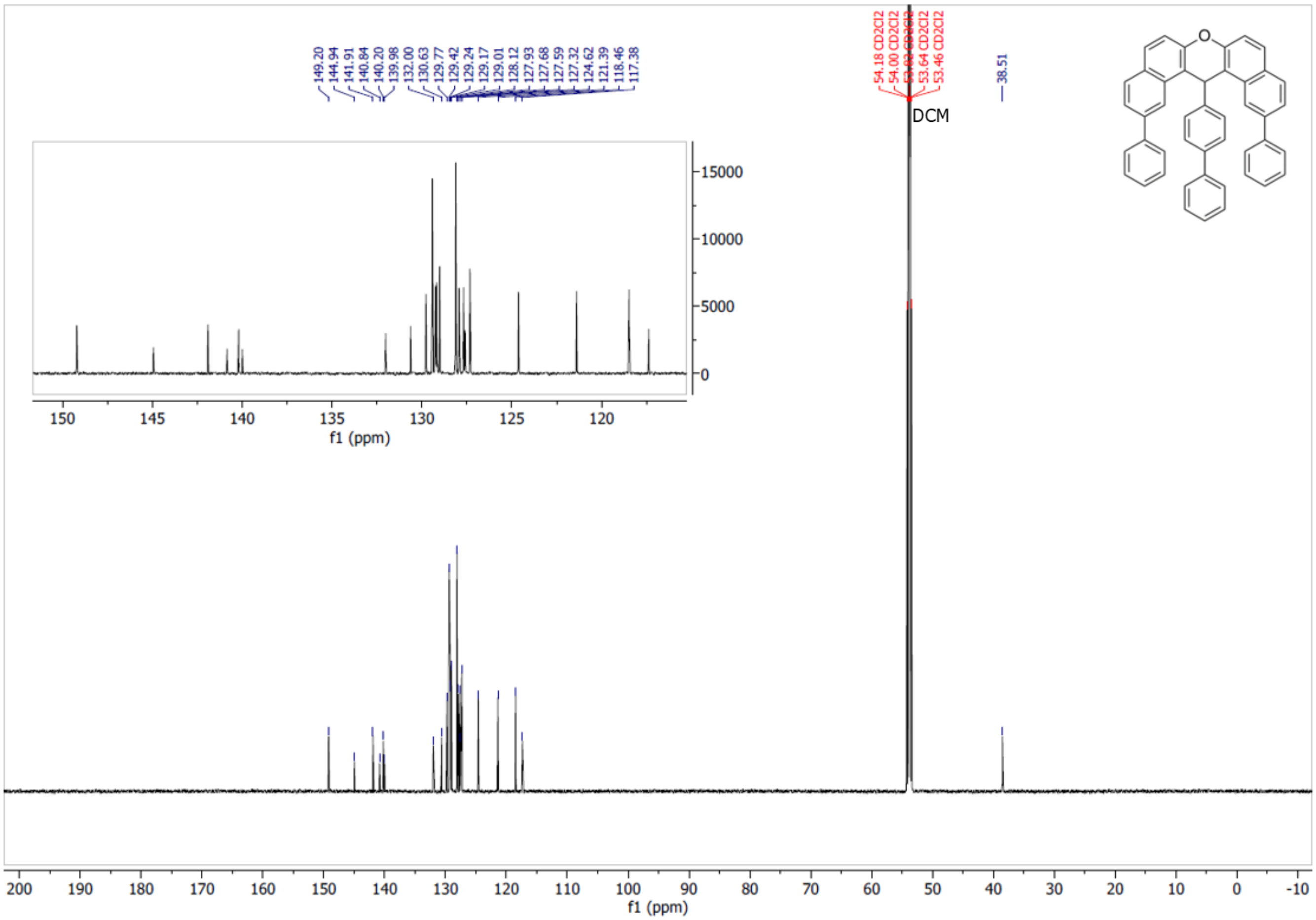

Figure S6. $^{13}C\{^{1}H\}$ NMR (151 MHz, CD_2Cl_2) spectrum of 14-([1,1'-biphenyl]-4-yl)-2,12-diphenyl-14H-dibenzo[a,j]xanthene (**4**) at 24 °C.

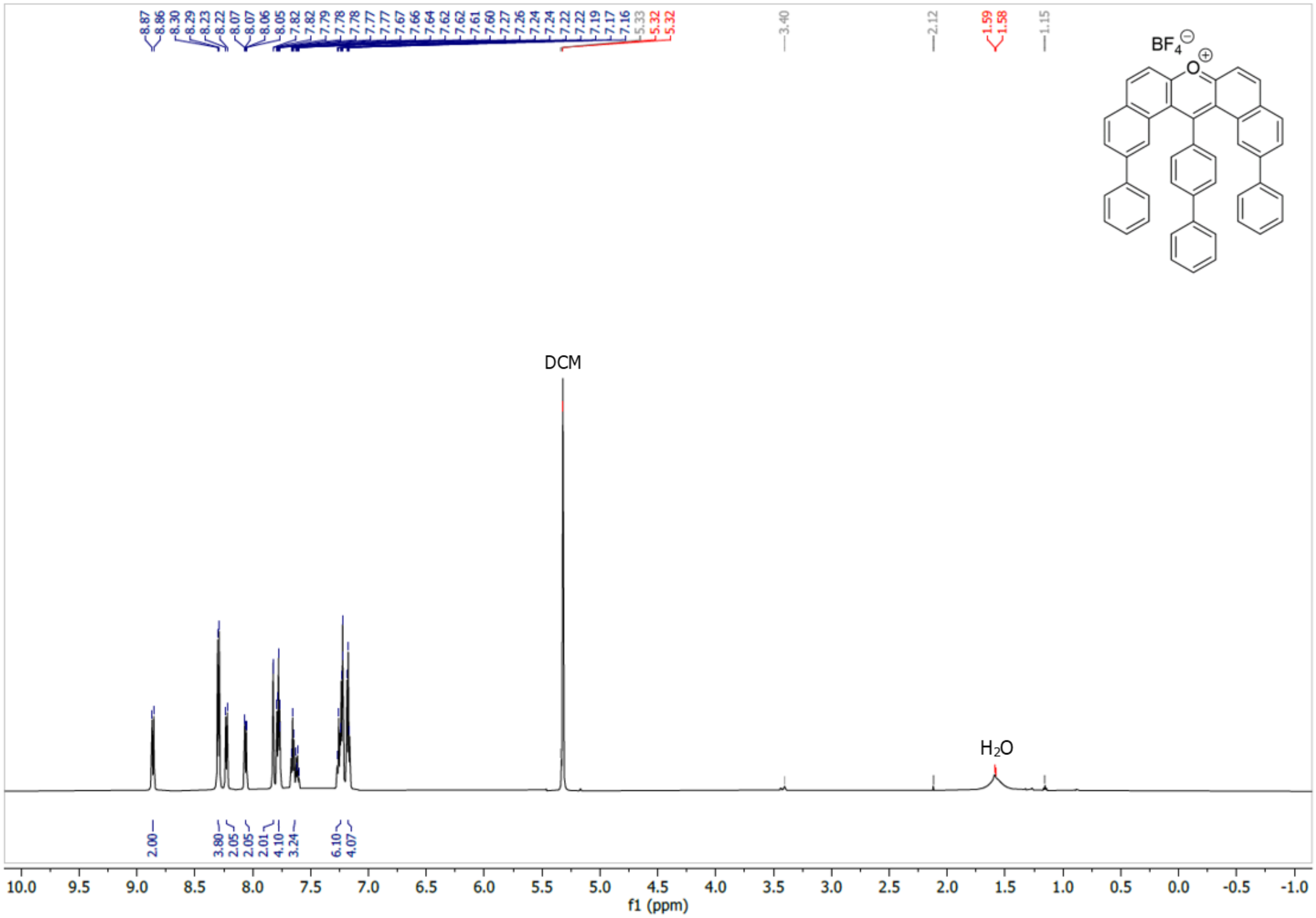

Figure S7. ^{1}H NMR (600 MHz, CD_2Cl_2) of 14-([1,1'-biphenyl]-4-yl)-2,12-diphenyldibenzo[*a*,*j*]xanthen-7-ium tetrafluoroborate (**6**) at 24 °C.

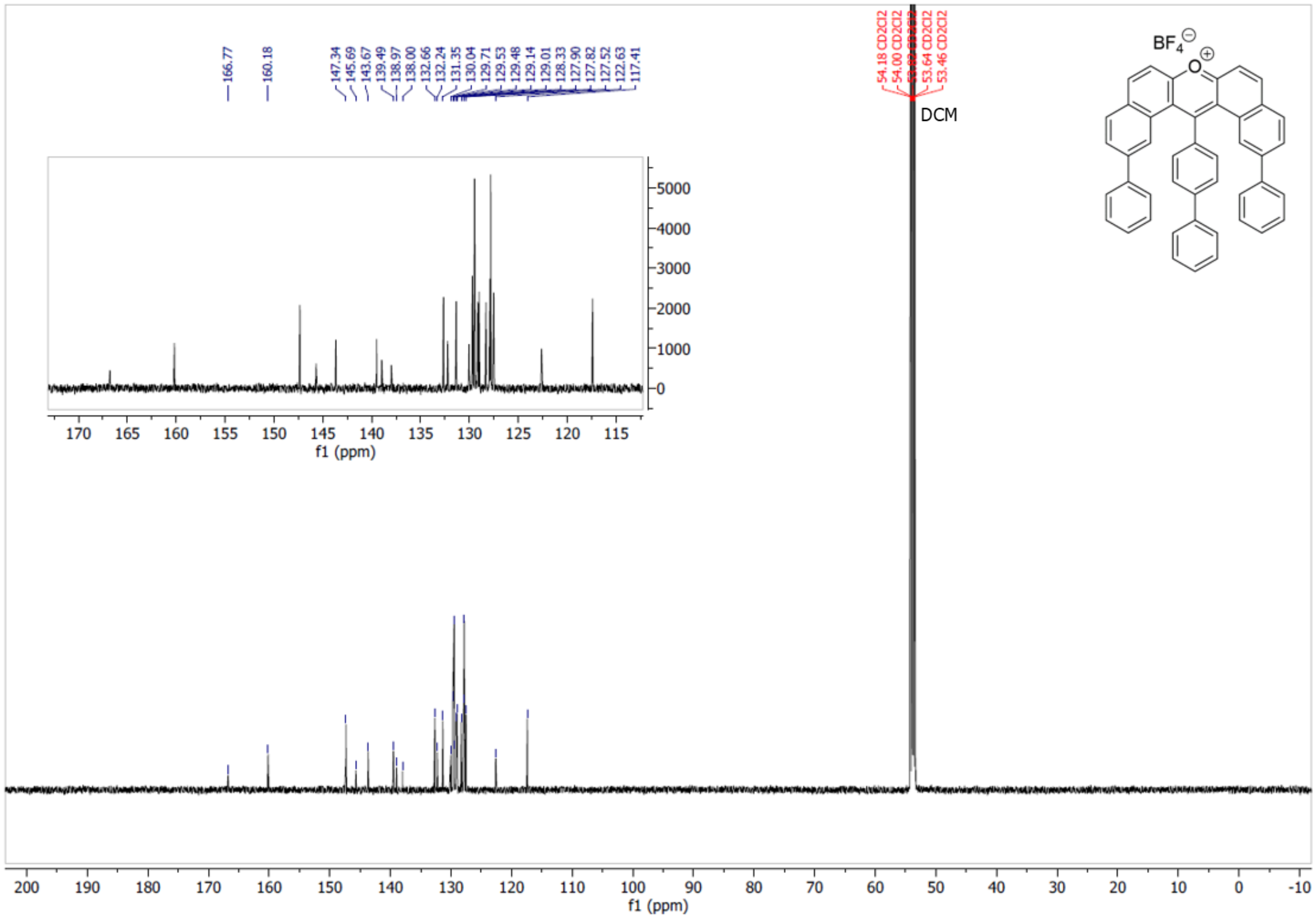

Figure S8. $^{13}C\{^{1}H\}$ NMR (151 MHz, CD_2Cl_2) spectrum of 14-([1,1'-biphenyl]-4-yl)-2,12-diphenyldibenzo[*a,j*]xanthen-7-ium tetrafluoroborate (**6**) at 24 °C.

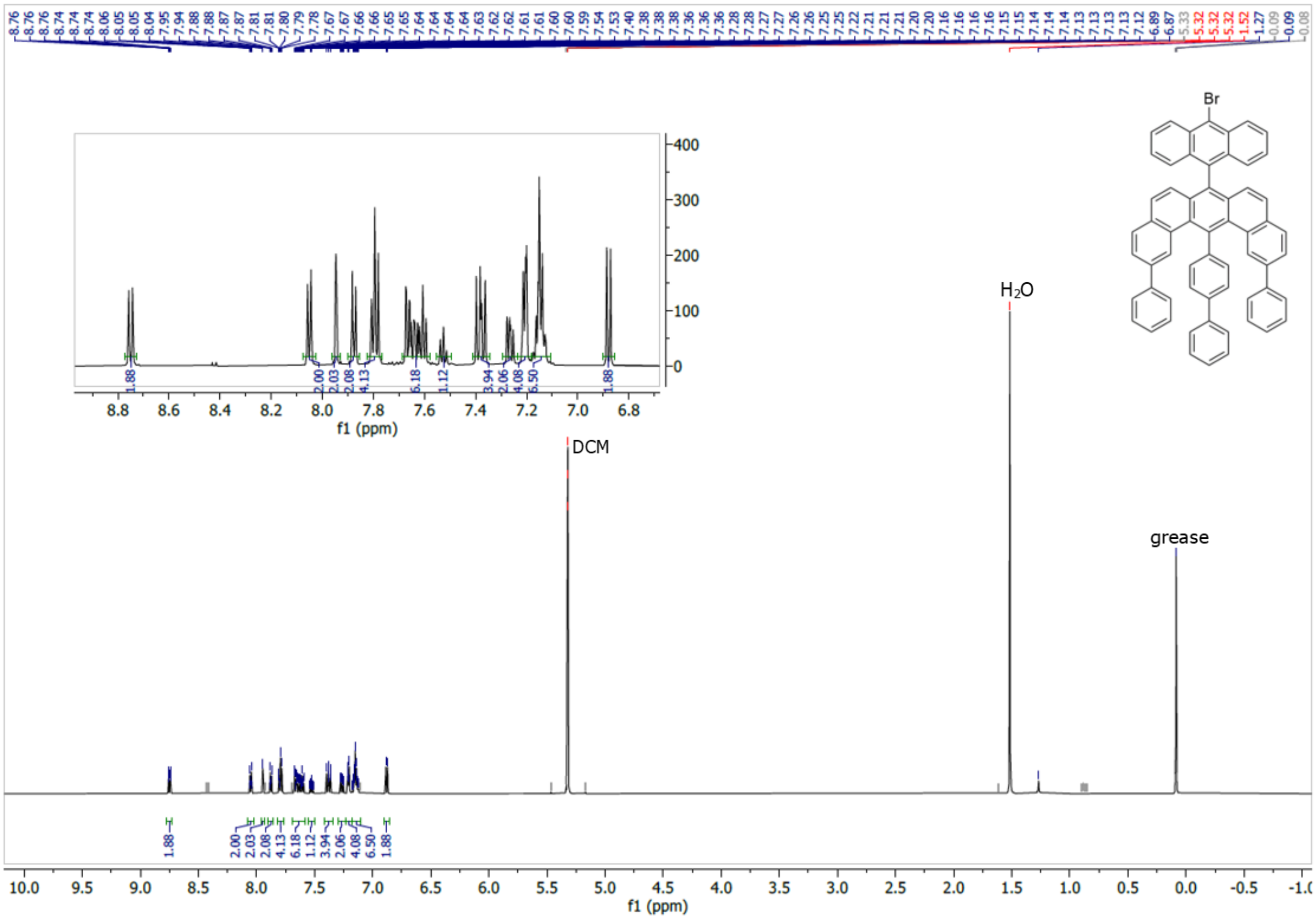

Figure S9. ^{1}H NMR (600 MHz, CD_2Cl_2) of 14-([1,1'-biphenyl]-4-yl)-7-(10-bromoanthracen-9-yl)-2,12 diphenylbenzo[m]tetraphenetetraphene (**1**) at 24 °C.

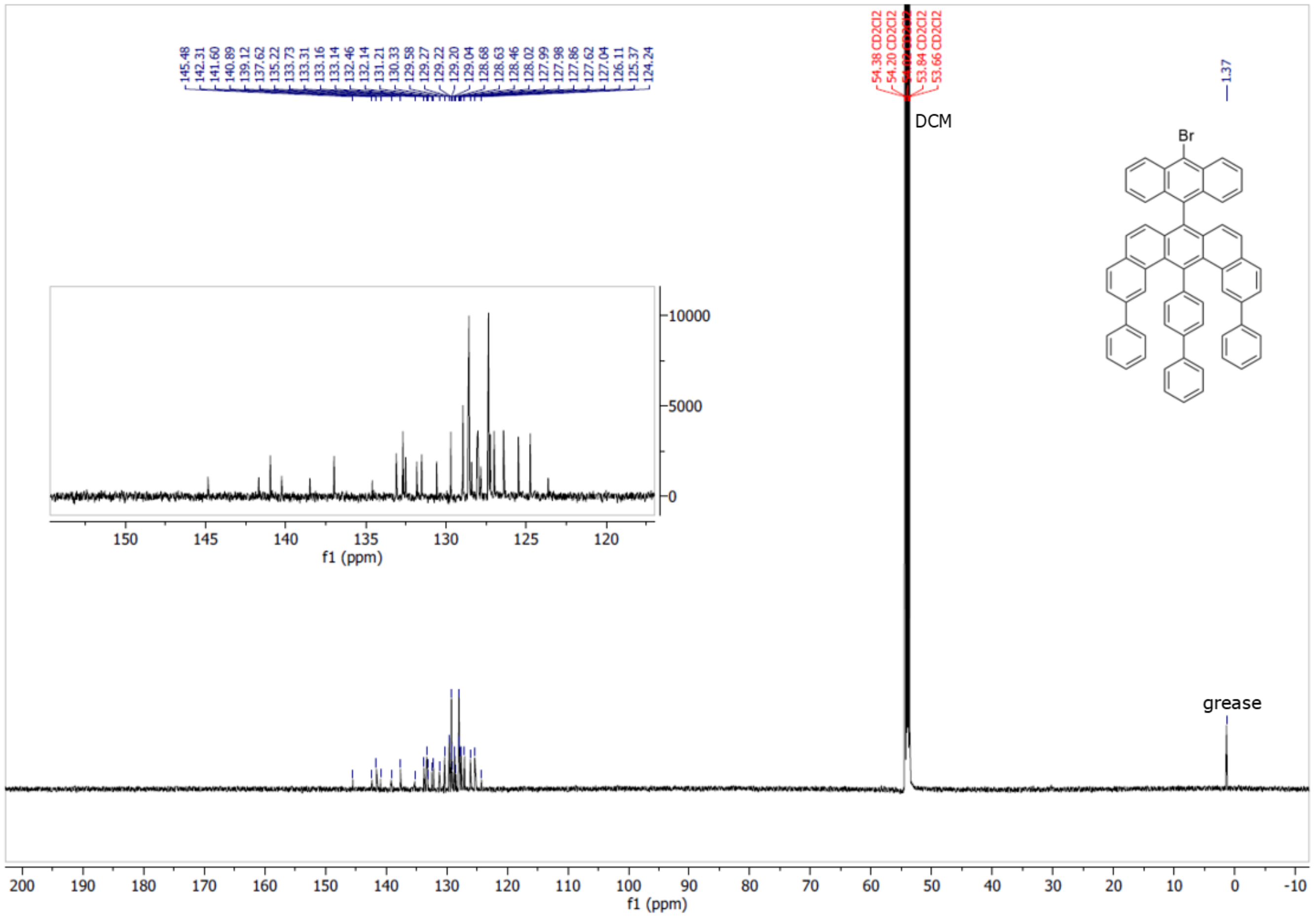

Figure S10. $^{13}C\{^{1}H\}$ NMR (151 MHz, CD_2Cl_2) spectrum of 14-([1,1'-biphenyl]-4-yl)-7-(10-bromoanthracen-9-yl)-2,12 di-phenylbenzo[*m*]tetraphenetetraphene (**1**) at 24 °C.

References:

(1) Giannozzi, P.; Baroni, S.; Bonini, N.; Calandra, M.; Car, R.; Cavazzoni, C.; Ceresoli, D.; Chiarotti, G. L.; Cococcioni, M.; Dabo, I.; Dal Corso, A.; de Gironcoli, S.; Fabris, S.; Fratesi, G.; Gebauer, R.; Gerstmann, U.; Gougoussis, C.; Kokalj, A.; Lazzeri, M.; Martin-Samos, L.; Marzari, N.; Mauri, F.; Mazzarello, R.; Paolini, S.; Pasquarello, A.; Paulatto, L.; Sbraccia, C.; Scandolo, S.; Sclauzero, G.; Seitsonen, A. P.; Smogunov, A.; Umari, P.; Wentzcovitch, R. M., QUANTUM ESPRESSO: a modular and open-source software project for quantum simulations of materials. *J. Phys. Condens. Mat.* **2009**, *21*, 395502.

(2) Giannozzi, P.; Andreussi, O.; Brumme, T.; Bunau, O.; Nardelli, M. B.; Calandra, M.; Car, R.; Cavazzoni, C.; Ceresoli, D.; Cococcioni, M.; Colonna, N.; Carnimeo, I.; Dal Corso, A.; de Gironcoli, S.; Delugas, P.; DiStasio, R. A.; Ferretti, A.; Floris, A.; Fratesi, G.; Fugallo, G.; Gebauer, R.; Gerstmann, U.; Giustino, F.; Gorni, T.; Jia, J.; Kawamura, M.; Ko, H. Y.; Kokalj, A.; Küçükbenli, E.; Lazzeri, M.; Marsili, M.; Marzari, N.; Mauri, F.; Nguyen, N. L.; Nguyen, H. V.; Otero-de-la-Roza, A.; Paulatto, L.; Poncé, S.; Rocca, D.; Sabatini, R.; Santra, B.; Schlipf, M.; Seitsonen, A. P.; Smogunov, A.; Timrov, I.; Thonhauser, T.; Umari, P.; Vast, N.; Wu, X.; Baroni, S., Advanced capabilities for materials modelling with QUANTUM ESPRESSO. *J Phys-Condens Mat* **2017**, *29*, 465901.

(3) Hamann, D. R., Optimized norm-conserving Vanderbilt pseudopotentials. *Physical Review B* **2013**, *88*, 085117.

(4) van Setten, M. J.; Giantomassi, M.; Bousquet, E.; Verstraete, M. J.; Hamann, D. R.; Gonze, X.; Rignanese, G. M., The PSEUDODOJO: Training and grading a 85 element optimized norm-conserving pseudopotential table. *Comput Phys Commun* **2018**, *226*, 39-54.
